# Pediatricians’ Knowledge of Emergency Management of Dental Injuries and Use of Mouthguards: A Cross-Sectional Survey

**DOI:** 10.3390/dj9120152

**Published:** 2021-12-15

**Authors:** Antonija Tadin, Danijela Delic, Ivana Kristina Delic Jukic, Kristina Gorseta, Lidia Gavic

**Affiliations:** 1 Department of Restorative Dental Medicine and Endodontics, School of Medicine, University of Split, 21000 Split, Croatia; lgavic@mefst.hr; 2 Department of Maxillofacial Surgery, University Hospital Split, 21000 Split, Croatia; 3 Department of Dental Medicine, Health Center of Split-Dalmatia County, 21000 Split, Croatia; danijela.stdelic@gmail.com; 4 Department of Nephrology, University Hospital Split, 21000 Split, Croatia; ikdelic@yahoo.com; 5 Department of Paediatric Dentistry, School of Dental Medicine, University of Zagreb, Gunduliceva 5, 10000 Zagreb, Croatia; gorseta@sfzg.hr

**Keywords:** dental trauma, mouthguards, multidisciplinary care, pediatric dentistry

## Abstract

Aim: Dental injuries are one of the most frequent oral health problems in children and adolescents. This study aimed to evaluate Croatian pediatricians’ knowledge and practice regarding dental injuries management and the use of mouthguards as means of protection against traumatic injuries. Materials and methods: A questionnaire-based cross-sectional survey conducted among 186 pediatricians examined their demographic characteristics, knowledge and experience with dental injuries. Student *t*-test or one-way ANOVA, with Tukey’s post-hoc were used to analyze obtained data (*p* ≤ 0.05). Results: The overall mean score of knowledge on the management of dental injuries was 3.6 ± 1.53 points. Male pediatricians (*p* = 0.016), those who witnessed dental injuries (*p* = 0.003), and those who had more than ten years of pediatric practice (*p* = 0.027) showed better knowledge. The results of multiple linear regression analysis demonstrated impact between pediatricians’ knowledge in the emergency treatment of traumatic dental injuries concerning the level of health care (practice settings) (β = −0.254, *p* = 0.002) and the number of patients treated by a pediatrician per day (β = −0.187, *p* = 0.030). Conclusion: Pediatricians have a low level of knowledge regarding the primary care of traumatic dental injuries. An additional course on dental injurie management should be recommended as part of the physician and pediatricians’ educational training.

## 1. Introduction

Dental injuries are one of the most common orofacial injuries. It has been reported that one-third of all preschool children, as well as one-fourth of all school-aged children and nearly one-third of adults, have sustained dental injuries [1,2,3]. Dental injuries range from simple dental tooth crown fractures to massive supportive tissue injuries such as avulsion. Regardless of the severity of the injury, it can affect children’s quality of life through a variety of functional, esthetic, speech, and psychological consequences [4,5,6].

Most dental injuries are considered emergencies that require immediate treatment. Delays in initial treatment may adversely affect the outcome of the affected tooth. Accurate assessment and prompt treatment of dental injuries are critical for the tooth’s prognosis. Physicians, particularly pediatricians, play an essential role in providing primary care after dental trauma, particularly in children with limited access to dental care [7].

Pediatricians are the custodians of the overall health of children. Since oral health is an integral part of overall health, they play an essential role maintaining of oral health by preventing and managing dental problems [8]. To date, numerous studies and systematic reviews have been conducted to determine the role of medical doctors and pediatricians in children’s oral health and to assess their knowledge, attitudes and professional practice experience. The majority of these studies have shown that physicians and pediatricians have limited knowledge and understanding of critical areas of dentistry, such as oral health and dental trauma management [9,10,11,12,13,14,15,16,17]. Their inadequate knowledge and practice experience result from insufficient education and a lack of oral health content in their educational process and training [8,12,14]. Since pediatricians are usually the first to whom parents or guardians bring their children with a health problem, they must have adequate knowledge to help their patients. That is especially important in areas with limited access to dental care, where pediatricians are the first and only contact point. And is particularly true for parents or guardians whose children have developmental disabilities, as these parents tend to have more confidence in pediatricians than in general dentists [8,18].

In Croatia, there are currently a small number of pediatric dentists, less than 50, and most of them work in primary care as general dentists. Only a few of them work with children in secondary and tertiary health care in Croatia’s three largest cities (Zagreb, Rijeka and Split). The number of pediatricians is higher, around 714, and they practice medicine in all country regions in primary, secondary and tertiary health care [19]. From this, it can be concluded that it is easier for children in Croatia to access pediatricians than specialists in pediatric dentistry. Thus, if a child sees a pediatrician for a dental health injury, they need to receive timely and valuable advice or care relative to their issue.

It is important to emphasize that given the scope of their work and knowledge, general dentists must pay sufficient attention to children and their problems. Possible barriers to caring for patients with dental trauma injuries can include insufficient knowledge, skills, experience, finances and confidence in treating dental trauma as well as uncooperative patients. As the quick and adequate reaction is essential for the prognosis of the tooth, the general dentist may decide to refer the child to a pediatric dentist for treatment of the tooth injury. However, due to the poor availability of pediatric dentists, it may take a long time for the child to get treated by one [20]. Therefore, it is necessary for all specialists who work with children, including pediatricians, to be adequately trained and informed on the possibilities of treatment and prevention of dental injuries. The present study aims to assess Croatian pediatricians’ attitudes and knowledge regarding the emergency management of dental injuries and the use of mouthguards in their prevention.

## 2. Materials and Methods

This questionnaire-based cross-sectional study was designed to assess pediatricians’ knowledge, attitudes, and experience regarding dental injuries and their management. The study was conducted in April 2018 during the annual symposium of pediatricians at Croatian Spring Pediatric School (Vis, Croatia). The study was approved by the Ethics Committee of the Faculty of Medicine, University of Split, Croatia. The study was conducted in full accordance with the ethical principles, including the World Medical Association Declaration of Helsinki (version 2008), and conducted according to the Reporting of Observational Studies’ guidelines in Epidemiology (STROBE).

The questionnaire included basic data about the study and indicated that participation was voluntary, anonymous, and free of charge. The inclusion criteria were medical doctors who specialized in pediatrics and had at least one year of clinical experience. The exclusion criteria were reluctance to participate in the study and retired pediatricians’ (age ≥ 65 years).

The minimum necessary sample size (n = 144) was calculated from the total number of pediatricians who attended the symposium with a 95% confidence interval, a 5% margin of error, and a 50% population proportion.

After an extensive literature analysis of studies examining pediatricians’ and physicians’ knowledge and attitudes on dental injuries, 30 closed response questions were selected and adapted to the Croatian population [13,15,16,17]. Two specialists (endodontics and pediatric dentistry specialist) reviewed the preliminary form version of the questionnaire and verified its appropriateness. The initial questions were drafted in English and then translated into Croatian. The questionnaire was pretested on ten pediatricians’ who were excused from primary study. The survey had good reliability, with a Cronbach’s alpha of 0.725.

The questions were divided into four parts. The first part consisted of issues related with demographic and professional data (age, gender, academic degree, workplace, number of years in pediatric practice, number of working hours per day, and number of patients per working day). The second part contained serval issues which formed the basis for the knowledge assessment. There were seven multiple-choice questions (six with one correct answer and one with multiple correct answers, but with a dichotomous scoring design) on dental trauma management [21]. The sum of correct answers was considered as the total knowledge of the respondents on the topic of dental injuries with a possible maximum score of seven. According to Bloom’s cut-off ranking, participants’ overall knowledge was classified as good if the score was between 80% and 100% (5.6–7 points), moderate if the score was between 60% and 79% (5.5–4.2 points), and poor if the score was less than 60% (<4.1 points) [22]. The third part of the questionnaire included questions about the participant’s personal experience, education and self-reported knowledge on dental injuries. The last part of the questionnaire consisted of questions about matters related to a dental mouthguard.

All statistical analyses were carried out using the IBM SPSS Statistics, version 25 (SPSS, IBM Corp, Armonk, NY, USA). The Kolmogorov–Smirnov test was used to assess compliance with the normal distribution of the dependent variable. Means and standard deviations were calculated for quantitative variables, while percentages and absolute numbers were used for presenting qualitative variables. Statistical analyses were conducted using the Student’s *t*-test or one-way ANOVA, with Tukey’s *post-hoc* test. To account for possible confounding, multiple linear regression analysis was used to determine a relationship between selected independent variables (age, gender, dental trauma training, witnessed or experienced dental injuries, academic degree) with the knowledge score (dependent variable). The significance level was set at *p* < 0.05.

## 3. Results

Questionnaires were distributed to 230 pediatricians’ who attended the symposium. 186 questionnaires were completed and returned to the authors (participation rate was 80%). The study included 78 men and 108 female pediatricians. The mean age of the participants was 47.2 ± 10.2 years. In Table 1 were shown the demographic and professional characteristics of the participants. Male pediatricians showed better knowledge about dental injury trauma compared to female respondents (*p* = 0.016). Considering the years spent in pediatric practice, participants with 11 to 20 years of experience showed the highest level of knowledge, compared to participants with professional experience both in the range of 1 to 10 years and those over 21 years (*p* = 0.027).

The distribution of correct responses regarding therapeutic procedures for tooth injuries at the accident site are presented in Table 2. About 60% of the participants answered that an erupted primary tooth can be replanted after tooth avulsion. Furthermore, 67.3% of the respondents did know how to hold an avulsed permanent tooth before reimplantation. In addition, 71% of the respondents were unfamiliar with the possibility of gently rinsing the tooth with tap water in case of tooth contamination before reimplantation at the site of the accident. Only 15.1% of pediatricians know that the best time to obtain professional assistance in the case of permanent tooth avulsion is within the first 30 min after the injury.

Table 3 shows the self-reported knowledge and experience of traumatic tooth injuries. Respondents expressed adequate but incomplete understanding of the subject in 58.6% of cases, based on self-assessed knowledge. In contrast, only 27.4% of the respondents expressed inadequate knowledge of traumatic tooth injuries. The majority of the respondents (94.1%) were not familiar with dental trauma, nor had they received the first aid training for such injuries during their studies or professional practice. The pediatricians’ who experienced dental trauma in their practice (3.9 ± 1.4 vs. 3.3 ±1.5, *p* ≤ 0.001) and who were willing to invest in further education on this topic (3.7 ± 1.4 vs. 3.1 ± 1.7, *p* = 0.008) showed a higher level of knowledge.

Table 4 shows the knowledge of pediatricians on the function and use of dental mouthguards. The higher level of knowledge in the management of dental injuries was shown by those pediatricians who knew the function of dental mouthguards’ (3.9 ± 1.5 vs. 3.2 ± 1.5, *p* ≤ 0.001). Similarly, better knowledge was shown among those pediatricians who recommend their patients to use a dental mouthguard during sports (3.8 ± 1.54 vs. 3.3 ± 1.5, *p* = 0.016).

**Figure 1:**
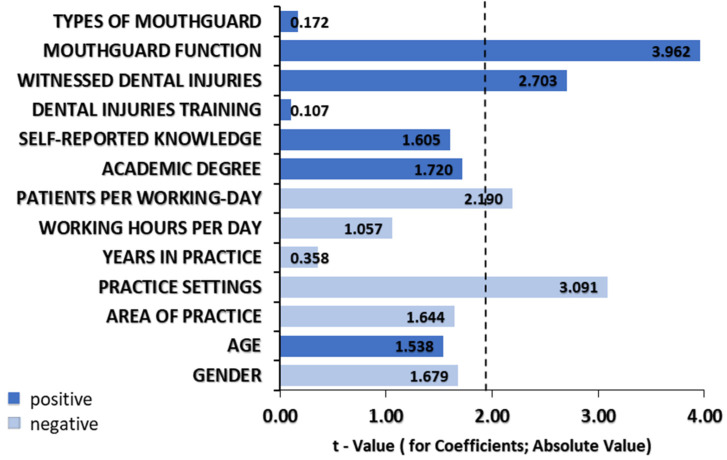
Multiple linear regression analysis. Significant dependence of measured overall dental injuries knowledge score with pediatrician’s demographical data and self-reported knowledge as possible predictors.

Figure 1 illustrates the association knowledge level in dental injury management with self-reported knowledge, demographic data, and attitude towards dental injuries. Knowledge score was significantly associated with pediatrician’s personal experience with dental injuries (β = 0.574, SE = 0.212, *p* = 0.008) and knowledge of dental mouthguard function (β = 0.883, SE = 0.223, *p* ≤ 0.001).

## 4. Discussion

This study aims to evaluate pediatricians’ knowledge of emergency management of dental injuries and the use of mouthguards as a preventive method. Since dentists rarely work in hospitals or emergency rooms in Croatia, it is imperative that pediatricians and physicians have adequate knowledge of therapeutic procedures for dental trauma. According to Flores et al. [23], the prognosis of the affected tooth depends on both the quality of the practitioner’s actions and the time that has elapsed between the accident and the treatment. There have been few studies worldwide, and only one in Croatia, that have investigated pediatricians’ knowledge of dental trauma [13,15]. When it comes to other medical personnel in non-dental professions, all studies conducted showed that physicians’ have inadequate knowledge of how to perform emergency procedures on the affected tooth [13,14,17,23].

The mean score of knowledge of emergency procedures for tooth injuries in our study was 3.6 ± 1.5 points (min. 0, max. 7), with three pediatricians having a score of zero (no correct answer) and four of them having the sum of seven (all correct answers). This is consistent with the pediatricians’ self-assessment of knowledge, were 58.6% (n = 109) of the respondents indicated that they had adequate knowledge, but it was described as incomplete. In addition, only 27.4% stated that they had inadequate knowledge (n = 51). The better mean score was obtained by male pediatricians (*p* = 0.016) and those pediatricians who have been in practice between 11 and 20 years (*p* = 0.027, Table 1). Although we cannot be sure, better knowledge in male respondents might be associated with the fact that many have been sports players who either have knowledge of or have first-hand experience of dental trauma related injuries. Only 5.9% of our respondents had attended courses on dental trauma, and they showed more excellent knowledge than their colleagues without relevant education, but without statistical significance (*p* = 0.351).

More than 75% of the respondents indicated that they were willing to increase their knowledge of dental trauma management. However, they showed better knowledge than their colleagues without the desire to learn (*p* = 0.008). Research conducted worldwide on medical personnel working in emergency departments showed that most of them had not received training in the management of dental trauma, 88% in the UK, 90% in Chile and 72% in Turkey [24,25,26]. These data indicate the need for a systematic approach to the education of medical personnel who may encounter with patients with dental injuries in their work.

The present study showed no statistical significance in pediatricians’ knowledge regarding their academic level of education (*p* = 0.738), level of health care (*p* = 0.086), and workplace (*p* = 0.105). The results of our study are consistent with those of Nikolic et al. [13] regarding pediatricians’ working in primary care and those working in hospitals.

About 45% of the respondents had witnessed dental trauma (n = 83), and they were most likely to witness dental avulsion (n = 76). These respondents also showed more excellent knowledge in managing dental injuries (*p* = 0.003, *p* = 0.001, respectively).

Pediatricians should be prepared to give appropriate advice to parents or guardians about first aid for avulsed teeth. They need to have general knowledge of eruption dates and patterns for deciduous and permanent teeth and the difference between a deciduous and a permanent tooth because primary teeth should not be replanted [9,27]. In our study, only 40.3% of the respondents knew that reimplantation of a deciduous tooth is not an option. In comparison, 59.1% of the respondents were aware that reimplantation is a therapy for the avulsion of a permanent tooth. Additionally, only 31.7% of the participants answered correctly that the avulsed tooth is held by its crown when restoring it in the alveolus. Somewhat different results were obtained by Nikolic et al. [13], where 60% and 68% knew the correct answers for deciduous and permanent tooth reimplantation, respectively, and 76% knew the right answer for tooth retention. In contrast, less than 50% of physicians in Turkey knew the correct method of tooth reimplantation [28]. An avulsed tooth that has come into contact with debris can be rinsed with cold water. However, the percentage of the respondents in our study who were aware of this was 29%

The outcome of reimplantation depends on several factors, such as the tooth storage medium and the replantation time, which can either accelerate or reduce the incidence of resorption or ankylosis. If immediate reimplantation is not possible for any reason, the tooth must be stored in a suitable medium until the arrival at the dentist [28,29]. In this study, almost all the respondents (94.6%) knew the answer for appropriate storage media such as saline, milk, the patient’s mouth or saliva, and a special tooth care medium. These media are necessary to preserve the vitality of the periodontal ligament during the extraoral period. In this study, 64.5% of the respondents considered saline as a suitable storage medium. In the study of Nikolic et al. [13] 54% of the respondents had an accurate opinion, unlike of Chanchala et al. [15] where 90% gave the same answer. Although saline is a suitable medium, it is not ideal due to its osmolarity [29]. Only 4.3% of the respondents chose a patient’s saliva as the answer, and 17.2% chose milk as the storage medium.

The time of reimplantation is critical to the long-term prognosis of the avulsed tooth. Reimplantation within 30 min has a 90% chance of success, while the chances of long-term retention become insignificant after two hours [28]. Unfortunately, only 15.1% of the pediatricians in this study were aware of the importance of an immediate response, referring the injured person to the dentist on time. Similar results were obtained in Aren et al. [28] survey on a sample of Turkish emergency workers.

Crown fractures of permanent tooth are the most usual type of dental injuries [30]. Fragment reattachment is one of the therapy options in the case of a fractured tooth [31]. Pediatricians must be aware of these possibilities and recommend that parents or guardians find the fragment of the broken tooth and keep it in a liquid medium. About 44% of the respondents knew this fact.

Mouthguards cover the teeth and surrounding mucosa to reduce the severity of dental injuries during sport activities. Various forms are available on the market, and custom-fitted mouthguards are considered to be the best option because of their convenience and better protection [32]. The use of mouthguards is recommended for all children and youth who participate in organized sport activities, especially contact sport (football, volleyball, basketball, handball, hockey, rugby, etc.) [33]. Majority of the respondents stated that they were both aware of the function of mouthguards and recommended them to their patients during sport. These respondents also showed a higher level of knowledge regarding emergency procedures for dental injuries. However, most of the respondents did not know the types of and differences between mouthguards (Table 4).

This study showed that pediatricians’ level of knowledge about dental injuries is insufficient when it comes to the implementation of therapeutic emergency procedures. This study shows that the management of dental injuries is neglected as a topic and that pediatricians’ education is inadequate. Indeed, within the curriculum of the five-year pediatric specialization in the Republic of Croatia, physicians are only required to complete one single-week course on pediatric dentistry in clinical hospital centers. Although pediatric dentistry is one of the courses offered, the management of dental injuries is not on the list of competencies that pediatricians must undertake during specialist training [34].

However, it is encouraging that many of pediatricians were aware of this inadequacy in their education and were willing to further develop their knowledge. Although it would be difficult to change the academic curriculum, this could be done by including the topic in various interdisciplinary seminars, postgraduate studies, symposia, etc. [8,10]. Various studies have confirmed the association of specific oral health educational interventions in pediatricians with improvement of their knowledge, attitudes and practices in that area. There are multiple ways to adequately educate pediatricians on oral health, such as modifying the pediatric training curriculum, creating evidence-based guidelines, using print media and providing in-service training through workshops and seminars. It is also important to create close teamwork between the medical and dental community by organizing scientific and clinical meetings [35,36,37]. Finally, pediatricians should be aware of the available evidence-based dental trauma management guidelines or literature, which they should refer to when consulting children with dental injuries and their parents. The main purpose of these guidelines is to give basic instructions for the immediate or urgent care of TDIs in order to provide the best possible care [27,31].

One strength of this study was that it was conducted among pediatricians in all regions of Croatia [13]. Additionally, the study identified several factors related to the knowledge and preventive practices of pediatricians regarding dental injuries. The methodology (paper-based, self-administered questionnaire) and good response rate were also advantages. The main limitation of this study was the potential selection bias caused by the voluntary nature of the survey and convenience sampling. We recommend further studies examining the knowledge and practice of pediatricians in other areas of oral health, especially when it comes to the prevention of malocclusion and oral diseases.

## 5. Conclusions

Even though pediatricians in Croatia showed insufficient knowledge of dental injury management, it is positive that they are aware and willing to learn. In addition to continuing education, the cooperation of all medical professionals in the cross-section is necessary to identify dental injuries in time and initiate urgent treatment measures accordingly.

## Figures and Tables

**Table 1:** Demographic and professional characteristic of respondents (n = 186).

Characteristic		Total n (%)	Knowledge Score Mean ± SD	*p* Value
Gender	Male	78 (41.9)	3.9 ± 1.4	0.016 *
	Female	108 (58.1)	3.4 ± 1.6	
Age group (years)	25–35	34 (18.3)	3.1 ± 1.6	0.224
	36–45	46 (24.7)	3.6 ± 1.5	
	46–55	56 (30.1)	3.6 ± 1.5	
	≥55	50 (26.9)	3.8 ± 1.5	
Academic degree	Doctor of Medicine	123 (66.1)	3.5 ± 1.5	0.738
	Master of Science	38 (20.4)	3.6 ± 1.5	
	Doctor of Philosophy	25 (13.4)	3.8 ± 1.7	
Area of practice	Northern—Croatia	60 (32.3)	3.5 ± 1.6	0.105
	Eastern—Croatia	13 (7.0)	3.9 ± 1.1	
	Southern—Croatia	73 (39.2)	3.8 ± 1.5	
	Western—Croatia	40 (21.5)	3.2 ± 1.5	
Practice setting	Primary care	92 (49.5)	3.6 ± 1.5	0.086
	Secondary care	43(23.1)	3.9 ± 1.6	
	Tertiary care	51 (27.4)	3.2 ± 1.4	
Number of years in practice	1–10	78 (41.9)	3.3 ± 1.5 ^a^	0.027 *
	11–20	57 (30.6)	3.9 ± 1.6 ^a^	
	≥21	51 (27.4)	3.6 ± 1.4	
Number of hours worked per workday	1–4	2 (1.1)	2.5 ± 0.7	0.561
	4–8	113 (60.8)	3.6 ± 1.4	
	≥8	71 (38.2)	3.5 ± 1.7	
Number of patients seen in a workday	<10	14 (7.5)	3.7 ± 1.7	0.368
	10–20	112 (60.2)	3.6 ± 1.5	
	21–50	35 (18.8)	3.7 ± 1.5	
	>50	25 (13.4)	3.1 ± 1.7	

Data are presented as whole numbers and percentages or mean ± SD. * Statistical significance was tested by Student *t*-test or One-way ANOVA. Statistical significance was set to *p* < 0.05. The same superscript lower letter indicated a statistical difference between groups based on *post-hoc* Tukey (^a^ *p* = 0.020). Abbreviation: SD—standard deviation.

**Table 2:** The frequency distribution (%) of pediatricians’ answers about emergency management of dental trauma injuries.

Question	Answer	Total n (%)
Can avulsed primary tooth be replanted?	*No*	75 (40.3)
	I do not know	90 (48.4)
	Yes	21 (11.3)
Can avulsed permanent tooth be replanted?	*Yes*	110 (59.1)
	I do not know	13 (7.0)
	No	63 (33.9)
During reimplantation, how would you hold an avulsed permanent tooth?	By the crown	59 (31.7)
	By the root	31 (16.7)
	By the whole tooth	20 (10.8)
	I do not know	74 (39.8)
If the avulsed permanent tooth was unable to be replanted, how would you store it until till reach the dentist?	*Special tissue culture solutions*	16 (8.6)
	*Saliva—mouth of child*	8 (4.3)
	*Milk*	32 (17.2)
	*Saline solution*	120 (64.5)
	Disinfection solutions	0 (0)
	Tap water	0 (0)
	Handkerchief	5 (2.7)
	Alcohol	3 (1.6)
	Ice	2 (1.1)
What is the ideal time to seek professional assistance in the case of permanent tooth avulsion?	*Immediately, within the first 30 min from the injuries*	28 (15.1)
	Within a few hours	83 (44.6)
	Within 24 h	45 (24.2)
	Next day	3 (1.6)
	Within few next days	1 (0.5)
	There are no limited restrictions	2 (1.1)
	I do not know	24 (12.9)
What is the best immediate treatment for an avulsed permanent tooth at site of accident?	*Wash the tooth with water and put it back into the socket*	54 (29.0)
	Wash the tooth with disinfection solution and put it back into the socket	16 (8.6)
	Scrub the tooth and put it back into the socket	85 (45.7)
	Put the tooth back into the socket without cleaning	8 (4.3)
	I do not know	23 (12.4)
Is it possible to provide reattachment of broken tooth fragment	*Yes*	81 (43.6)
	No	55 (29.5)
	I do not know	50 (26.9)

Data are presented as whole numbers and percentages. Correct answers are italicized.

**Table 3:** Pediatrician’s knowledge and experience with dental injures (n = 186).

Question		Total n (%)	Knowledge Score Mean ± SD	*p* Value
Self-reported knowledge about dental injuries	Insufficient	51 (27.4)	3.7 ± 1.7	0.060
	Sufficient but incomplete	109 (58.6)	3.6 ± 1.5	
	Comprehensive	26 (14.0)	3.7 ± 1.5	
Dental injuries and first aid training during academic or professional education	No	175 (94.1)	3.5 ± 1.5	0.351
	Yes	11 (5.9)	4.0 ± 1.3	
Willing to attend training on dental injuries in future	No	44 (23.7)	3.0 ± 1.7	0.008 *
	Yes	142 (76.3)	3.7 ± 1.4	
Witnessed dental injures	No	103 (55.4)	3.3 ± 1.5	0.003 *
	Yes	83 (44.6)	3.9 ± 1.4	
Witnessed dental injures—avulsion	No	110 (59.1)	3.3 ± 1.5	0.001 *
	Yes	76 (40.9)	4.0 ± 1.4	
Witnessed dental injures—tooth luxation (lateral luxation, intrusion, extrusion)	No	170 (91.4)	3.7 ± 1.5	0.904
	Yes	16 (8.6)	3.6 ± 1.7	
Witnessed dental injures—tooth fracture	No	141 (75.8)	3.5 ± 1.5	0.186
	Yes	45 (24.2)	3.8 ± 1.5	
Self-reported knowledge about dental injuries first aid—avulsion	No	83 (44.6)	3.2 ± 1.5	0.001 *
	Yes	103 (55.4)	3.9 ± 1.4	
Self-reported knowledge about dental injuries first aid—tooth luxation	No	179 (96.2)	3.5 ± 1.5	0.216
	Yes	7 (3.8)	4.3 ± 1.5	
Self-reported knowledge about dental injuries first aid—tooth fracture	No	122 (65.6)	3.5 ± 1.5	0.375
	Yes	64 (34.4)	3.7 ± 1.5	

Data are presented as whole numbers and percentages or mean ± SD. * Statistical significance was tested by Student’s *t*-test or one-way ANOVA. Statistical significance was set to *p* < 0.05. Abbreviation: SD—standard deviation.

**Table 4:** Pediatrician’s knowledge regarding dental mouthguard (n = 186).

Questions		Total n (%)	Knowledge Score Mean ± SD	*p* Value
Do you know mouthguard function?	No	83 (44.6)	3.1 ± 1.5	0.001 *
	Yes	103 (55.4)	3.9 ± 1.5	
Do you recommend to your patients the usage of mouthguard?	No	88 (47.3)	3.3 ± 1.5	0.016 *
	Yes	98 (52.7)	3.8 ± 1.5	
Do you know the types of mouthguards?	No	176 (94.6)	3.6 ± 1.5	0.500
	Yes	10 (5.4)	3.9 ± 1.4	
Do you know the difference between custom and off-the-shelf mouthguards?	No	4 (2.2)	2.2 ± 1.5	0.192
	Yes	47 (25.3)	3.7 ± 1.6	
	I do not know	135 (72.6)	3.6 ± 1.5	
How much usage of mouthguard can prevent or reduce sports-related dental injuries?	10%	4 (2.2)	3.2 ± 1.5	0.880
	20%	25 (13.4)	3.4 ± 1.5	
	40%	91 (48.9)	3.6 ± 1.3	
	60%	66 (35.5)	3.6 ± 1.8	

Data are presented as whole numbers and percentages or mean ± SD. * Statistical significance was tested by Student’s *t*-test or one-way ANOVA. Statistical significance was set to *p* < 0.05. Abbreviation: SD—standard deviation.

## Data Availability

Not applicable.
